# Supplementary material for: Exploring the Association between Oxygen Concentration and Life Expectancy in China: A Quantitative Analysis
Source: Int J Environ Res Public Health. 2023 Jan 8;20(2):1125. doi: 10.3390/ijerph20021125 (PMC9859324; doi:10.3390/ijerph20021125)
Supplement: Supplementary file 1 [file ijerph-20-01125-s001.zip › ijerph-2015861-supplementary.pdf]

S1 Appendix

Supplementary materials to "Exploring the Association between Oxygen Concentration and Life Expectancy in China: A Quantitative Analysis"

Table S1. Materials and sources

**Variables**	**Period**	**Data location**	**Spatial / Temporal resolution**	**Source**
Life expectancy (years)	2015	33 provinces	Province-level /1 year	[20]
		Taiwan		A news report released by the Global Times (http://www.taiwan.cn/taiwan/tw_SocialNews/201609/t20160930_11582973.htm)
	2012-2018	39 municipalities	Municipality-level /1 year	Official websites of the municipal health commissions, health statistics bureaus and Centers for Disease Control and Prevention
Meteorological data (pressure (kPa), sunshine (h), wind speed (m/s), relative humidity (%), and temperature (℃))	2000-2015	(1)31 provinces<br>(2)39 municipalities	Point-level / 1day	National Meteorological Data Center (http://data.cma.cn/)
		Macau	Province-level /1 day	Earth Geography and Meteorological Bureau (https://www.smg.gov.mo/zh)
		Hong Kong		Hong Kong Observatory (https://www.hko.gov.hk/sc/index.html)
	1991-2000	Taiwan		Central Meteorological Bureau of the Ministry of Communications (https://www.cwb.gov.tw/V8/C/)
Number of health technicians	2015	31 provinces	Province-level /1 year	China Statistical Yearbook 2016 (http://www.stats.gov.cn/tjsj/ndsj/2016/indexch.htm)
	2014	Hong Kong		National Bureau of Statistics
	2014	Macau		
	2015	Taiwan		Taiwan Statistical Yearbook 2018 (https://www.doc88.com/p-6771713009489.html)
	2015	39 municipalities	Municipality-level /1 year	"2015 National Economic and Social Development Statistical Bulletin" of each region, obtained from China Statistical Information Network (http://www.tjcn.org/tjgb/)

GDP per capita (￥)	2015	31 provinces	Province-level /1 year	China Statistical Yearbook 2016 (http://www.stats.gov.cn/tjsj/ndsj/2016/indexch.htm)
		Hong Kong		Hong Kong Statistical Yearbook 2016 (https://www.censtatd.gov.hk/en/data/stat_report/product/B1010003/att/B10100032016AN16B0100.pdf)
		Macau		Statistics and Census Bureau of Macau Special Administrative Region (https://www.dsec.gov.mo/zh-MO/)
		Taiwan		A news report released by the official Xinhua news agency (http://m.haiwainet.cn/middle/345691/2016/0129/content_29600835_1.html)
		39 municipalities	Municipality-level /1 year	"2015 National Economic and Social Development Statistical Bulletin" of each region, obtained from China Statistical Information Network (http://www.tjcn.org/tjgb/)
Number of students in university (college and above), Number of students in high school, Number of students in middle school, Number of students in primary school, Populations not attending school, Number of populations aged six years and over	2015	34 provinces	Province-level /1 year	China Statistical Yearbook 2016 (http://www.stats.gov.cn/tjsj/ndsj/2016/indexch.htm)
		39 municipalities	Municipality-level /1 year	"2015 National Economic and Social Development Statistical Bulletin" of each region, obtained from China Statistical Information Network (http://www.tjcn.org/tjgb/)
Age-	2017	34 provinces	Province-level /1	[21]

standardized years of life lost per 100000 population for the top 20 level 3 causes in China			year	
Number of permanent residents at the end of the year	2015	34 provinces	Province-level /1 year	China Statistical Yearbook 2016 (http://www.stats.gov.cn/tjsj/ndsj/2016/indexch.htm)
		39 municipalities	Municipality-level /1 year	"2015 National Economic and Social Development Statistical Bulletin" of each region, obtained from China Statistical Information Network (http://www.tjcn.org/tjgb/)

Table S2. Summary of variables

**Variables**	**Provincial data (n = 34)**	**Municipal data (n = 39)**
Life expectancy (years) [Φ]	76.48±3.76	79.35±2.62
Oxygen concentration (mmHg) [Γ]	155.18 (143.99–158.07)	158.34 (152.63–159.21)
Percentage of higher education (%) [Γ]	1.93 (1.67-2.32)	2.65 (1.68–5.91)
Health technicians per 1000 people [Γ]	5.60 (5.14–6.30)	7.17 (6.23–8.84)
GDP per capita (￥)[Γ]	45715.50 (36762.25–72736.75)	87833.00 (56972.00–110351.00)
Sunshine (h) [Φ]	6.80±1.46	6.25±1.58
Temperature (℃) [Φ]	17.37±5.21	17.61±4.65
Wind speed (m/s) [Γ]	3.30 (2.78–4.41)	2.70 (2.46–3.37)
Relative humidity (%) [Γ]	71.25 (58.67–77.78)	74.12 (69.62–77.06)

[Φ] Summarized by mean ± standard deviation. [Γ] Summarized by median (lower quartile - upper quartile).

Table S3. The Moran's *I* index of spatial effects of life expectancy between provinces and municipalities

**Residual spatial variation of life expectancy**	**Moran's *I***	***P* value**	**Expectation**	**Variance**
**Provincial data**	0.472	<0.001	-0.03	0.010
**Municipal data**	0.106	0.238	-0.04	0.044

Table S4. Results of multiple linear regression

	β (95% CI)	Standardized β	P value	Adjusted R^2
**Provincial data**				0.734
constant	50.213 (42.304, 58.121)	-	<0.001*	
Oxygen concentration (mmHg)	0.146 (0.095, 0.196)	0.557	<0.001*	
GDP per capita (10^4 ￥) #	0.862 (-0.067, 1.792)	0.229	0.068	
Health technicians per 1,000 people	0.757 (0.211, 1.304)	0.340	0.008*	
**Municipal data**				0.745
constant	53.713 (43.684, 63.742)	-	<0.001*	
Oxygen concentration (mmHg)	0.170 (0.116, 0.224)	0.584	<0.001*	
GDP per capita (10^4 ￥) #	1.415 (0.924, 1.906)	0.540	<0.001*	
Health technicians per 1,000 people	0.342 (0.014, 0.669)	0.207	0.041*	
Average years of education (years)	-0.313 (-0.907, 0.083)	-0.154	0.118	

Abbreviation: 95% CI, 95% confidence interval. * Indicates P<0.05. # GDP per capita were standardized.

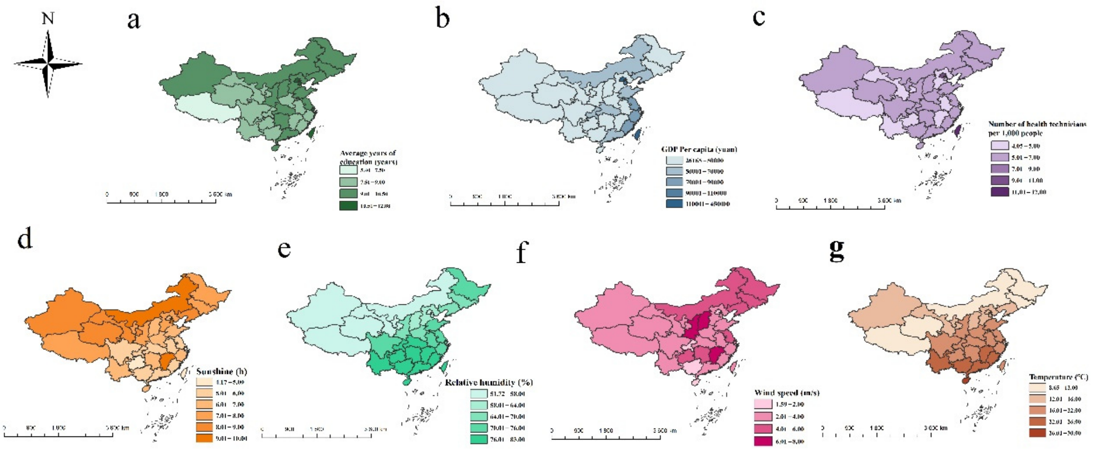

Figure S1. Distribution maps of potential influencing at the provincial level in China. (a) Average years of education in 34 provinces; (b) GDP per capita in 34 provinces; (c) Number of health technicians per 1,000 people in 34 provinces; (d) Sunshine in 34 provinces; (e) Relative humidity in 34 provinces; (f) Wind speed in 34 provinces; (g) Temperature in 34 provinces.

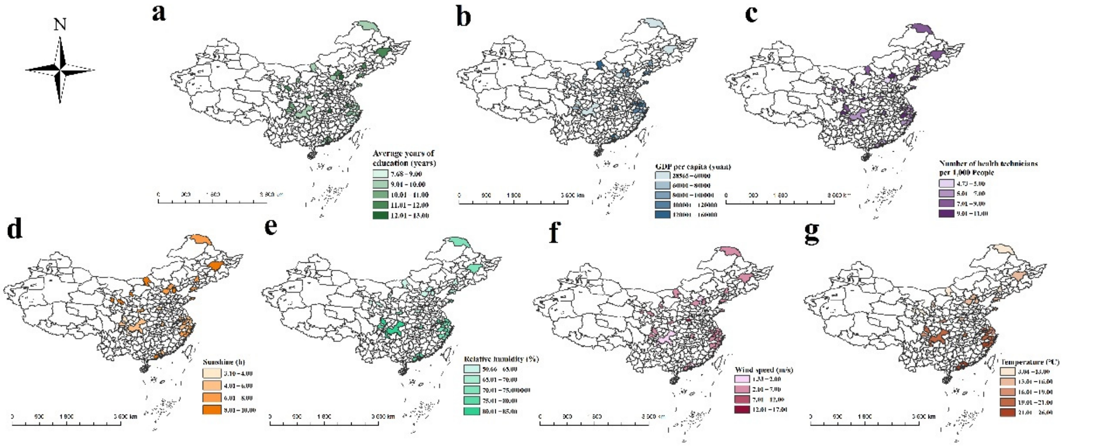

Figure S2. Distribution maps of potential influencing at the municipal level in China. (a) Average years of education in 39 municipalities; (b) GDP per capita in 39 municipalities; (c) Number of health technicians per 1,000 people in 39 municipalities; (d) Sunshine in 39 municipalities; (e) Relative humidity in 39 municipalities; (f) Wind speed in 39 municipalities; (g) Temperature in 39 municipalities.
